# Comparison of *Shigella* GMMA and glycoconjugate four-component formulations in animals

**DOI:** 10.3389/fmolb.2023.1284515

**Published:** 2023-11-16

**Authors:** Roberta Di Benedetto, Francesca Mancini, Valentina Caradonna, Maria Grazia Aruta, Carlo Giannelli, Omar Rossi, Francesca Micoli

**Affiliations:** ^1^ GSK Vaccines Institute for Global Health (GVGH), Siena, Italy; ^2^ Department of Life Sciences, University of Trieste, Trieste, Italy

**Keywords:** GMMA, glycoconjugate, *Shigella*, multicomponent, vaccine, O-antigen

## Abstract

Shigellosis is leading bacterial cause of diarrhea with high prevalence in children younger than 5 years in low- and middle-income countries, and increasing number of reports of *Shigella* cases associated to anti-microbial resistance. No vaccines against *Shigella* are still licensed, but different candidates based on the O-antigen portion of lipopolysaccharides are in clinic. Generalized Modules for Membrane Antigens (GMMA) have been proposed as an alternative delivery system for the O-antigen, and a 4-component vaccine candidate (altSonflex1-2-3), containing GMMA from *S. sonnei* and *S. flexneri* 1b, 2a and 3a is being tested in a phase 1/2 clinical trial, with the aim to elicit broad protection against the most prevalent *Shigella* serotypes. Here, the 4-component GMMA vaccine candidate has been compared to a more traditional glycoconjugate formulation for the ability to induce functional antibodies in mice and rabbits. In mice, in the absence of Alhydrogel, GMMA induce higher IgG antibodies than glycoconjugates and stronger bactericidal titers against all *Shigella* serotypes. In the presence of Alhydrogel, GMMA induce O-antigen specific IgG levels similar to traditional glycoconjugates, but with a broader range of IgG subclasses, resulting in stronger bactericidal activity. In rabbits, GMMA elicit higher functional antibodies than glycoconjugates against *S. sonnei*, and similar responses to *S. flexneri* 1b, 2a and 3a, independently from the presence of Alhydrogel. Different O-antigen based vaccines against *Shigella* are now in clinical stage and it will be of particular interest to understand how the preclinical findings in the different animal models translate in humans.

## 1 Introduction

Shigellosis is leading bacterial cause of diarrhea in low- and middle-income countries, particularly in young children under five years of age (Kotloff et al., 2018; Kotloff et al., 2019). As antibiotic resistance to *Shigella* is increasing (Puzari et al., 2018; Ranjbar and Farahani, 2019; Raso et al., 2023), this pathogen has been identified as a priority for the development of a vaccine (World Health Organisation, 2022). No vaccines are currently licensed against *Shigella*, but different candidates are under development (Mani et al., 2016; MacLennan et al., 2022), many based on the O-antigen (OAg) portion of lipopolysaccharide (LPS) (Cohen et al., 2022; Martin and Alaimo, 2022; Phalipon and Mulard, 2022), recognized as key target for protective immunity (Robbins et al., 1992; Cohen et al., 2019).

We are developing a four-component *Shigella* vaccine based on Generalized Modules for Membrane Antigens (GMMA) as alternative delivery system for the OAg (Mancini et al., 2021b; Micoli et al., 2022b; Rossi et al., 2023).

GMMA are Outer Membrane Vesicles (OMVs) from Gram-negative bacteria genetically engineered to increase yields and produced through a simple and robust detergent-free manufacturing process (Berlanda Scorza et al., 2012; Gerke et al., 2015; Kis et al., 2019). GMMA combine multivalent display of saccharides and proteins in their native outer membrane environment, with the presence of immunostimulatory molecules, such as LPS, lipoproteins and peptidoglycans, and nanoparticle size (Micoli and MacLennan, 2020; Piccioli et al., 2022). Additional mutations can be easily introduced to modify the lipid A component and reduce endotoxicity to minimize GMMA ability to promote reactogenicity (Rossi et al., 2014; Mancini et al., 2021b).

A mono-component *S. sonnei* GMMA vaccine candidate has been first evaluated in phase 1/2 clinical trials in healthy adults from *Shigella* non-endemic (EU) and endemic (Kenya) countries showing to be immunogenic and well tolerated (Launay et al., 2017; Obiero et al., 2017; Launay et al., 2019; Micoli et al., 2021b; Kapulu et al., 2022). However, the vaccine failed to confer protection against shigellosis in a Controlled Human Infection Model (CHIM) study (Frenck et al., 2021). Thus, an improved version of *S. sonnei* GMMA has been designed to have increased OAg density with respect to the previous construct and this new generation component has been formulated with GMMA from three *S. flexneri* serotypes (1b, 2a and 3a) in a four-component formulation called altSonflex1-2-3, aiming to provide broad protection against the most prevalent *Shigella* serotypes (Livio et al., 2014; Citiulo et al., 2021). The altSonflex1-2-3 vaccine is currently being evaluated in a Phase 1/2 clinical trial to test safety and immunogenicity in 9-month infants and to identify optimal dosing and schedule (Micoli et al., 2022b; Rossi et al., 2023).

Here, we compare head to head, in animal models, the GMMA approach to the more classical glycoconjugation approach for an OAg-based vaccine against *Shigella*. Indeed, conjugation of OAg to appropriate carrier proteins is a well established approach for improving immunogenicity, providing T-cell stimulation to the OAg which contains only B-cell epitopes (Schneerson et al., 1980). This results in enhanced memory response, IgG class-switching and improved immunogenicity in infants as well as in adults (Costantino et al., 2011; Rappuoli, 2018; Berti and Micoli, 2020). A *S. sonnei* OAg glycoconjugate was developed at the U.S. National Institutes of Health (NIH) (Robbins et al., 1995; Passwell et al., 2010; Barel and Mulard, 2019) showing 74% protection in adults after a single dose (Cohen et al., 1997) but failing to protect the younger population (Passwell et al., 2010). A well-defined *S. flexneri* 2a synthetic glycoconjugate vaccine, developed at Institut Pasteur has shown to be safe and immunogenic in a phase 1 study in adults after a single dose (Cohen et al., 2021), and is now being tested in phase 2 and CHIM trials (Phalipon and Mulard, 2022). Moreover, LimmaTech Biologics produced a bioconjugate against *S. flexneri 2a*, Flexyn2a, which proved to be immunogenic in phase 1 (Riddle et al., 2016) and protective against severe shigellosis in a CHIM study (Martin and Alaimo, 2022). These results supported the development of a four-component formulation, made of bioconjugates of *S. sonnei* and *S. flexneri* 2a, 3a and 6, that is currently tested in an age-descending dose-finding phase 2 trial in Kenya to evaluate vaccine safety and immunogenicity (Martin and Alaimo, 2022).

Results from this work contribute to understanding potential differences between traditional conjugates and GMMA as delivery systems for *Shigella* OAg.

## 2 Materials and methods

### 2.1 Preparation and characterization of GMMA

*Shigella* GMMA were produced from following strains: *S. sonnei* 53G Δ*tolR*:*kan* Δ*virG*:*nadAB* Δ*msbB2*:*cat* Δ*msbB*:*erm*, *S. flexneri* 1b Stansfield Δ*tolR*:*frt* Δ*msbB1a*:*frt* Δ*msbB1b*:*frt*, *S. flexneri* 2a 2457T Δ*tolR*:*kan*, Δ*msbB*:*cat*, and *S. flexneri* 3a 6885 Δ*tolR*:*kan*, Δ*msbB*:*cat*, and purified as previously described (Rossi et al., 2023). Purified GMMA were characterized for total protein content by micro BCA (Thermo Scientific, Waltham, MA, USA), total OAg amount by high-performance anion-exchange chromatography/pulsed amperometric detection (HPAEC-PAD) and OAg to protein ratio was calculated. GMMA size was estimated by dynamic light scattering (dls), OAg molecular size was determined by size exclusion-high-performance liquid chromatography (HPLC-SEC) after acetic acid extraction (Micoli et al., 2022a).

### 2.2 Preparation and characterization of glycoconjugates

OAg were extracted from *S. sonnei* (*S. sonnei* 53G Δ*tolR*:*kan* Δ*virG*:*nadAB*), *S. flexneri* 1b (strain *S. flexneri* 1b Stansfield Δ*tolR*:*frt* Δ*msbB1a*:*frt* Δ*msbB1b*:*frt*), 2a (strain *S. flexneri* 2a 2457T Δ*tolR*:*kan*, Δ*msbB*:*cat*), 3a (strain *S. flexneri* 3a 6885 Δ*tolR*:*kan*) GMMA, purified and fully characterized as previously described (Micoli et al., 2021a). OAg were independently activated with 1-cyano-4-dimethylaminopyridine tetrafluoroborate (CDAP) using the following procedure (Shafer et al., 2000): 100 mg/ml CDAP in acetonitrile was added to 9 mg/ml OAg in 2M NaCl with a 1.5:1 weight ratio CDAP/OAg. Soon after, 0.3M NaOH was added to reach pH 9. After 3 min, CRM_197_ was added to the solution in a CRM_197_/OAg 1:1 weight ratio with final concentration of 5 mg/ml. The reaction was mixed for 2 h at room temperature maintaining the pH at nine by adding 0.3M NaOH. At the end, 2M glycine at pH nine was added in a weigth ratio of 7.5:1 glycine/OAg to quench the reaction. The solution was mildly mixed over night at room temperature.

*S. sonnei* OAg conjugate was purified by size exclusion chromatography on a 1.6 cm × 60 cm Sephacryl S-300 column (Cytiva Life Sciences, Marlborough, MA, USA; formerly GE Healthcare Life Sciences) eluted at 0.5 ml/min in Phosphate Buffer Saline (PBS). Fractions at higher molecular weight that did not overlap with free OAg and free CRM_197_ were collected. *S flexneri* OAg conjugates were purified by hydrophobic interaction chromatography (HIC) on a Phenyl HP column (Cytiva Life Sciences, Marlborough, MA, USA; formerly GE Healthcare Life Sciences), loaded in 20 mM NaH_2_PO_4_ 3M NaCl at pH 7.2. The purified conjugates were eluted in 20 mM NaH_2_PO_4_ at pH 7.2 and the collected fractions were exhanged against PBS by Amicon Ultra (Merck, Darmstadt, Germany) 30 kDa cut-off.

Purified conjugates were characterized by micro BCA (Thermo Scientific, Waltham, MA, USA) and HPAEC-PAD (Micoli et al., 2014; Giannelli et al., 2020; Micoli et al., 2022a) for total protein and total OAg content respectively and the OAg to protein ratio was calculated. Free polysaccharide was separated through reverse phase-solid phase extraction (SPE) using Vydac C4 SPE cartridges and quantified by HPAEC-PAD (Angela et al., 2005). Conjugates formation was verified by HPLC-SEC, comparing the conjugates with unconjugated CRM_197_ (Stefanetti et al., 2014).

### 2.3 GMMA and glycoconjugates formulation

Four-component GMMA with Alhydrogel formulation was prepared by adsorbing *S. sonnei* and *S. flexneri* 1b, 2a and 3a GMMA in NaCl 154 mM NaH_2_PO_4_ 10 mM pH 6.5 on 0.7 mg/ml (Al^3+^) Alhydrogel at the final concentration of 120 μg/ml total OAg (30 μg/ml each OAg). Further dilutions for immunogenicity studies were performed with Alhydrogel diluent (0.7 mg/ml Al^3+^ in NaCl 154 mM NaH_2_PO_4_ 10 mM pH 6.5). Four-component GMMA without Alhydrogel formulation was prepared by diluting *S. sonnei* and *S. flexneri* 1b, 2a and 3a GMMA in NaCl 154 mM NaH_2_PO_4_ 10 mM pH 6.5 at the final concentration of 12 μg/ml total OAg (3 μg/ml each OAg). Further dilutions were performed with NaCl 154 mM NaH_2_PO_4_ 10 mM pH 6.5.

*S. sonnei* and *S. flexneri* 1b, 2a and 3a glycoconjugates were first diluted in NaCl 154 mM at the final concentration of 12 μg/ml total OAg (3 μg/ml each OAg). Further dilutions were performed with NaCl 154 mM (formulations without Alhydrogel) or 0.7 mg/ml Al^3+^ in NaCl 154 mM (formulations with Alhydrogel).

### 2.4 *In vivo* studies

“GSK is committed to the Replacement, Reduction and Refinement of animal studies (3Rs). Non-animal models and alternative technologies are part of our strategy and employed where possible. When animals are required, application of robust study design principles and peer review minimises animal use, reduces harm and improves benefit in studies”.

Mouse and rabbit studies were performed at the GSK Animal Facility (Siena, Italy), in compliance with the relevant guidelines (Italian D. Lgs. n. 26/14 and European directive 2010/63/UE) and the institutional policies of GSK. The animal protocols were approved by the Italian Ministry of Health (project No. 1140/2020-PR, approval date 18/11/2020).

Female, 5 weeks old CD1 mice (8 per group) were vaccinated intraperitoneally (i.p.) with 200 µL of formulated antigens at study day 0 and 28. Approximately 100 µL bleeds (50 µL serum) were collected at day -1 (pooled sera) and at day 27 (individual sera), with final bleed at day 42.

Female New Zealand White rabbits Crl:KBL(NZW) (8 per group) were vaccinated intramuscularly (i.m.) with 500 µL of formulated antigens at study day 0 and 28 or 0 and 84. Sera were collected on study days -1 (pooled), 27, 42, 83 (all animals) and 98 (animals who only received immunization at day 84). Maximum volume of blood was sampled according to ethic’s recommendations.

### 2.5 Sera analyses

Sera collected at different time points were analysed by enzyme-linked immunosorbent assay (ELISA). ELISA plates were coated as follow: *S. sonnei* LPS at the concentration of 0.5 μg/ml in PBS, *S. flexneri* 1b OAg at the concentration of 2 μg/ml in Carbonate Buffer, *S. flexneri* 2a OAg at the concentration of 5 μg/ml in Carbonate Buffer. *S. flexneri* 3a OAg at the concentration of 1 μg/ml in PBS. Plates were blocked with PBS milk 5%, and incubated with the sera diluted 1:100, 1:4,000 and 1:160,000 in PBS-Tween 0.05% 0.1% BSA (for mouse sera) or PBS milk 5% (for rabbit sera). Bound antibodies were then detected using an enzyme-labelled secondary antibody (anti-mouse or anti-rabbit IgG-alkaline phosphatase, anti-mouse IgG1, IgG2a, IgG2b, IgG3 and IgM-alkaline phosphatase, codes reported in Supplementary Table S1) in PBS-Tween 0.05% 0.1% BSA. The presence of immunoreacting anti-*S. sonnei* LPS/*S. flexneri* 1b, 2a, 3a OAg antibodies was detected by addition of substrate solution and formation of a yellow color detected by absorbance at 405 nm subtracted by the absorbance at 490 nm. The samples were tested in comparison to calibrated mouse or rabbit anti-antigens specific reference standard sera. Results were expressed in ELISA units/mL determined relative to the reference serum. One ELISA unit equals the reciprocal of the dilution of the reference serum that yields an OD of one in the assay.

Individual serum samples collected at day 42 (mice and rabbits), 83 and 98 (rabbits) were also tested against bacterial strains (*S. sonnei* 53G *virG*:*cat* (Caboni et al., 2015), *S. flexneri* 1b, Stansfield NTCT five strain; *S. flexneri* 2a, 2457T strain and *S. flexneri* 3a, 6885 strain) in SBA based on luminescent readout as previously described (Necchi et al., 2017; Mancini et al., 2022). Results of the assay were expressed as the IC50, the reciprocal serum dilution that resulted in a 50% reduction of luminescence and thus corresponding to 50% growth inhibition of the bacteria present in the assay. Conditions used were optimized for each bacterial strain in terms of percentage of Baby Rabbit Complement used and buffer of the assay as previously reported (Citiulo et al., 2021). GraphPad Prism seven software was used for curve fitting and IC50 determination. A titer equal to half of the first dilution of sera tested was assigned to titers below the minimum measurable signal (i.e., 50).

### 2.6 Statistics

Statistical analysis was performed using GraphPad Prism 7. Mann-Whitney two-tailed test was used to compare the immune response elicited by two different formulations compared at same antigen dose. Wilcoxon test matched-pairs signed rank two-tailed test was performed to compare the response induced by the same formulation at different timepoints.

## 3 Results

### 3.1 Characterization of GMMA and glycoconjugates

With the intent of comparing in animal models GMMA and glycoconjugation approaches for an OAg-based vaccine against *Shigella*, OAg glycoconjugates were produced making use of CRM_197_, one of the most extensively used carrier proteins in licensed vaccines (Micoli et al., 2018a). GMMA were produced and purified as previously reported (Rossi et al., 2023).

**FIGURE 1:**
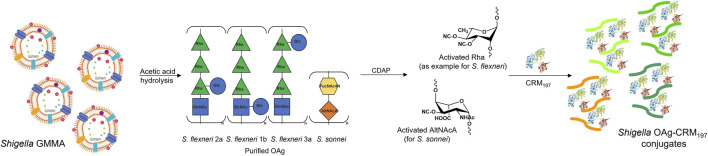
Purified *S. sonnei*, *S. flexneri* 1b, 2a and 3a OAg extracted from GMMA by acetic acid hydrolysis were conjugated to CRM_197_ through random activation of hydroxyl groups along the polysaccharide chain using the cyanilating agent CDAP, followed by linkage with lysines on the carrier protein.

*S. sonnei*, *S. flexneri* 1b, 2a and 3a OAg were extracted from corresponding GMMA (Micoli et al., 2021a) and independently linked to CRM_197_. Molecular size distribution of OAg used for conjugation and of OAg populations present on corresponding GMMA are reported in Table 1. Only in the case of *S. sonnei*, GMMA had an additional population at higher molecular weight compared to the OAg used for conjugation, corresponding to the group 4 capsule (G4C) (Gasperini et al., 2021). Also to be noted that GMMA present lipooligosaccharide chains at around 2 kDa, with core only or core plus few OAg repeating units, that were instead removed during OAg purification for glycoconjugates. Hydroxyl groups along the OAg chain were randomly activated using the CDAP cyanilating agent, followed by conjugation with lysine residues on CRM_197_ through formation of isourea linkages (Shafer et al., 2000) (Figure 1). Conjugates formation was verified by HPLC-SEC, revealing the presence of higher molecular weight peaks compared to the protein alone with no detectable unreacted CRM_197_. *S. sonnei* OAg conjugate was purified by size exclusion chromatography while *S. flexneri* conjugates were purified by HIC. All conjugates were characterized by an OAg to protein weight ratio in the range of 0.37–0.5, with <25% free saccharide. To be noted that OAg to protein ratio was similar for *S. sonnei* GMMA (0.29), but higher and close to one for all *S. flexneri* GMMA (Table 1). GMMA have particulate size in the range 82.5–160.4 nm as verified by dls analysis, while glycoconjugates size was estimated in the range 47.3–329.5 kDa by HPLC-SEC using dextrans as standards (Table 1).

**TABLE 1:** Main characteristics of GMMA and glycoconjugates compared in animal studies.

	Total OAg/protein w/w ratio		OAg molecular size distribution		Size	
	GMMA	Glyco-conjugate	GMMA	Glyco-conjugate	GMMA	Glyco-conjugate
*S. sonnei*	0.29	0.38	234 kDa (G4C); 19.2 kDa; 2.2 kDa	19.3 kDa	160.4 nm (0.16 PdI)	47.3 kDa
*S. flexneri* 1b	1.18	0.37	13.8 kDa; 1.7 kDa	13.6 kDa	108.9 nm (0.18 PdI)	82.1 kDa
*S. flexneri* 2a	1.11	0.41	47.1 kDa; 14.2 kDa; 1.8 kDa	35.8 kDa; 14.4 kDa	109.7 nm (0.14 PdI)	209.3 kDa
*S. flexneri* 3a	1.17	0.50	15.5 kDa; 1.8 kDa	15.2 kDa with 70.1 kDa shoulder	82.5 nm (0.17 PdI)	329.5 kDa

### 3.2 GMMA and glycoconjugates compared in mice

GMMA and glycoconjugates were formulated, without and with Alhydrogel, in corresponding four-component formulations and tested in mice at four different OAg doses, ranging from 9.4 to 600 ng of total OAg (2.3–150 ng of each OAg).

**FIGURE 2:**
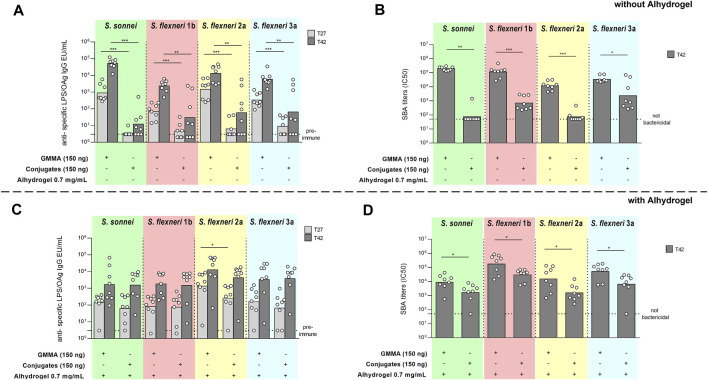
Comparison of GMMA and glycoconjugate 4-component formulations in mice in the absence **(A,B)** or presence **(C,D)** of Alhydrogel. CD1 mice were immunized intraperitoneally (i.p.) at day 0 and 28 with 9 ng, 37 ng, 150 ng and 600 ng total OAg dose. If present, concentration of Alhydrogel was 0.7 mg/ml (Al3+). Results at 150 ng total OAg dose are reported here as representative of the other doses. Sera were analyzed by **(A,C)** ELISA for LPS-specific (*S. sonnei*) or OAg-specific (*S. flexneri*) total IgG (EU/mL) and **(B,D)** serum bactericidal activity (SBA) assay for bactericidal titers expressed as IC50. Summary graphs of geometric mean units (bars) and individual levels (dots) are reported. *p < 0.05; **p < 0.01; ***p < 0.001.

In the absence of Alhydrogel, GMMA elicited significantly higher IgG antibodies than glycoconjugates at all the doses tested (data not shown), both 27 days after first injection or 14 days after the second injection, which was given at day 28. Results reported in Figure 2A are from the selected dose of 150 ng total OAg, representative of what observed at all tested doses. The reduced OAg-specific IgG response observed for glycoconjugates compared to GMMA was associated to lower bactericidal activity against all *Shigella* serotypes (Figure 2B).

When adsorbed on Alhydrogel, 27 days after the first injection, a higher anti-OAg IgG response was observed against *S. flexneri* 2a in mice immunized with GMMA vs*.* glycoconjugates, while comparable responses were induced against *S. sonnei*, *S. flexneri* 1b and 3a and post two against all *Shigella* serotypes (Figure 2C). However, in terms of sera functionality, GMMA elicited statistically significantly higher SBA titers against all *Shigella* serotypes compared to glycoconjugates (Figure 2D).

Upon second immunization GMMA boosted the response either when adsorbed on Alhydrogel or not, while glycoconjugates only when Alhydrogel-adjuvanted.

To further evaluate differences between the immune responses elicited by GMMA and glycoconjugate formulations, a deeper characterization of the quality of the humoral response was performed through analysis of IgG subclasses and IgM in sera from immunizations with 150 ng total OAg per dose.

**FIGURE 3:**
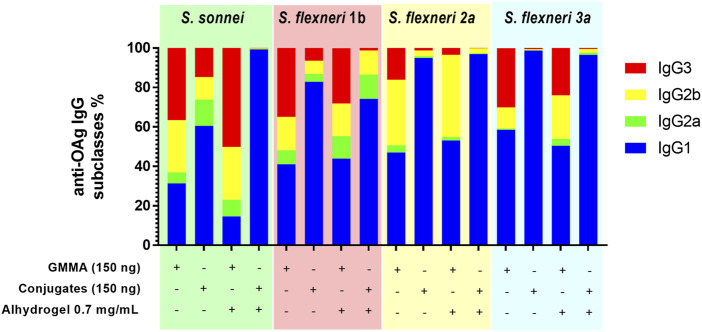
Characterization of the quality of the humoral response elicited by GMMA and glycoconjugates in mice. CD1 mice were immunized i. p. at day 0 and 28 with 150 ng total OAg dose in absence or presence of Alhydrogel. Anti-OAg-specific IgG subclasses have been evaluated at day 42. Relative percentage of each specific IgG subclass in respect to total IgG (ratio of geometric means) is reported in different colors in the bar plot for each formulation tested.

In the absence of Alhydrogel, for all serotypes, GMMA induced not only significantly higher IgG1, but also significantly higher IgG2, IgG3 and IgM than glycoconjugates. In the presence of Alhydrogel, GMMA and glycoconjugates elicited comparable levels of IgG1. Also IgG2a, IgG2b and IgM elicited by *S. sonnei*, *S. flexneri* 1b and 3a GMMA and glycoconjugates were similar, while were higher for *S. flexneri* 2a GMMA vs. glycoconjugates. IgG3 induced by GMMA were always higher than those elicited by glycoconjugates, except for *S. flexneri* 1b formulations eliciting a similar response (Supplementary Figure S1, Supplementary Table S2). In general, glycoconjugates elicited predominantly IgG1, while GMMA induced a broader range of IgG subclasses (Figure 3).

### 3.3 GMMA and glycoconjugates in rabbits

Same GMMA and glycoconjugate formulations were also compared in rabbits, at the dose of 600 ng of total OAg (150 ng of each OAg). In this study, same immunization scheme already used with mice was tested, but with intramuscular injection.

**FIGURE 4:**
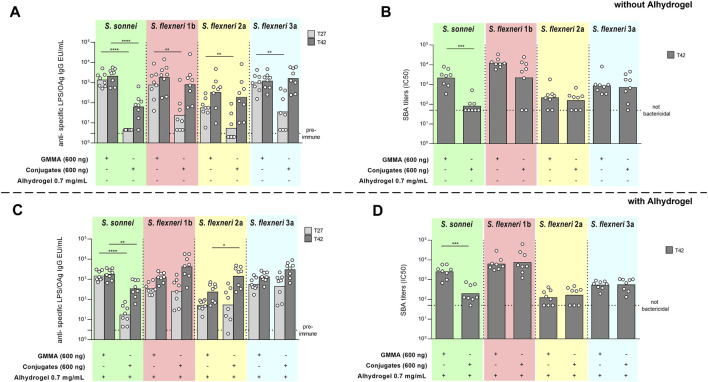
Comparison of GMMA and glycoconjugates 4-component formulations in rabbits in the absence **(A,B)** or presence **(C,D)** of Alhydrogel. New Zealand rabbits were immunized intramuscolarly (i.m.) at day 0 and 28 with 600 ng total OAg per dose. If present, concentration of Alhydrogel was 0.7 mg/ml (Al3+). Sera were analyzed by **(A,C)** ELISA for LPS-specific (*S. sonnei*) or OAg-specific (*S. flexneri*) total IgG (EU/mL) and **(B,D)** SBA for bactericidal titers expressed as IC50. Summary graphs of geometric mean units (bars) and individual levels (dots) are reported. *p < 0.05; **p < 0.01; ***p < 0.001.

A stronger *S. sonnei* LPS-specific total IgG response was elicited by GMMA with respect to glycoconjugates at all time points investigated, both without and with Alhydrogel (Figures 4A,C). SBA titers paralleled the IgG response (Figures 4B,D).

For all *S. flexneri* serotypes, the anti-OAg IgG response elicited by GMMA was significantly higher than glycoconjugates only post one and in the absence of Alhydrogel (Figure 4A **)**, whereas at day 42 the responses induced were similar with the exception of higher anti-*S. flexneri* 2a OAg IgG response elicited by glycoconjugates with respect to GMMA in the presence of Alhydrogel (Figure 4C). No differences were observed in terms of bactericidal titers, evaluated at day 42, without or with Alhydrogel, for all serotypes (Figures 4B,D).

### 3.4 Different immunization schedules tested in rabbits

Two different immunization schedules were compared in rabbits with GMMA and glycoconjugates formulated on Alhydrogel. Animals were immunized at day 0 and 1 month (day 28) or 3 months (day 84) later.

**FIGURE 5:**
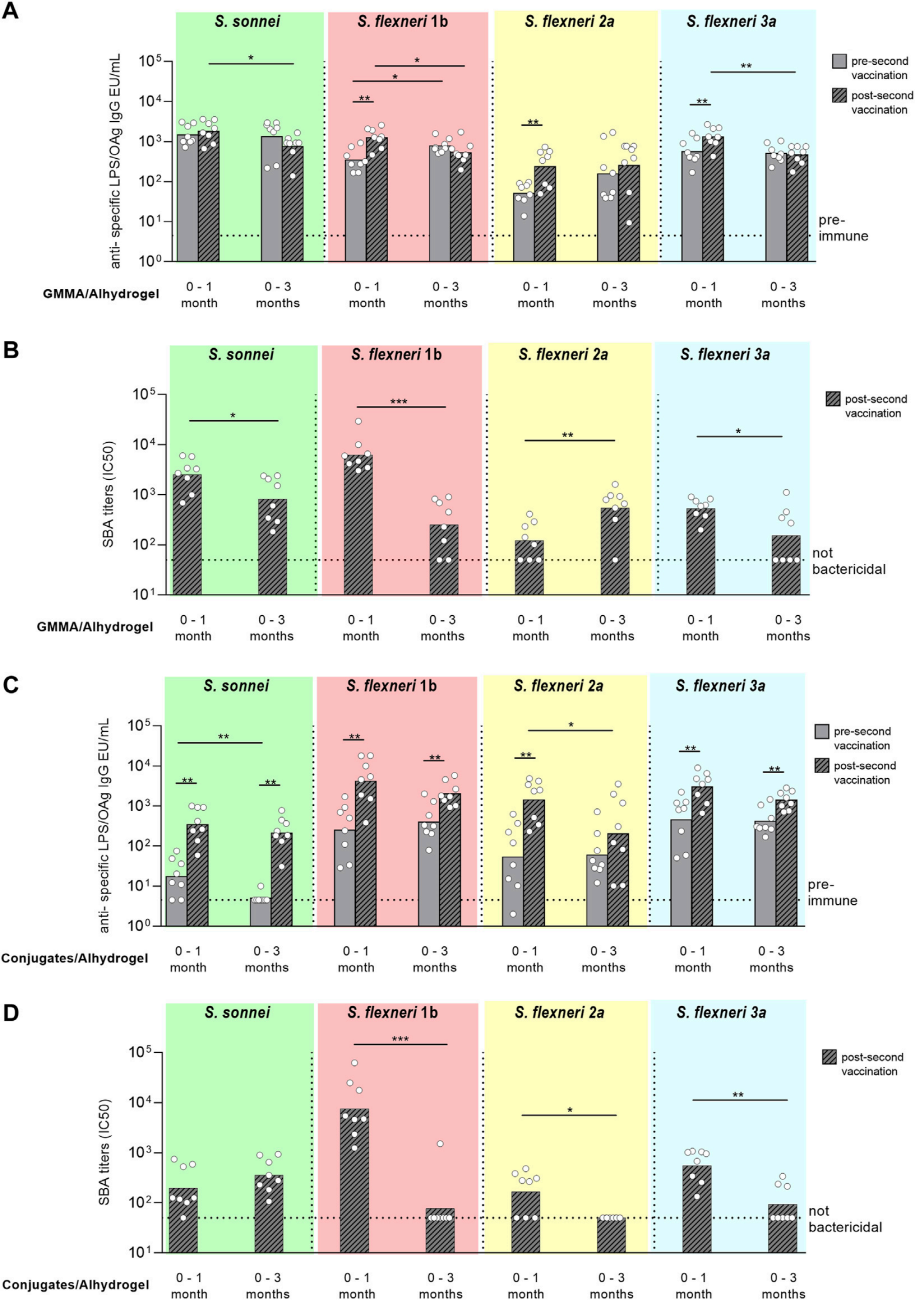
Comparison of two different immunization schemes for GMMA and glycoconjugate 4-component formulations in rabbits. New Zealand rabbits were immunized i. m. at day 0 and 28 (0–1 month) or 0 and 84 (0–3 months) with 600 ng total OAg per dose in presence of 0.7 mg/ml of Alhydrogel (Al3+). Sera were analyzed by **(A,C)** ELISA for LPS-specific (*S. sonnei*) or OAg-specific (*S. flexneri*) total IgG (EU/mL) pre second vaccination (at day 27 or 83) and post second vaccination (at day 42 or 98) and by **(B,D)** SBA for bactericidal titers expressed as IC50 post second vaccination only. Summary graphs of geometric mean units (bars) and individual levels (dots) are reported. *p < 0.05; **p < 0.01; ***p < 0.001.

Pre-second vaccination, the anti-OAg specific IgG responses elicited by GMMA were similar for all serotypes at day 27 (pre-second immunization at day 28) or 83 (pre-second vaccination at day 84), except for *S. flexneri* 1b with higher IgG titers induced at day 83 vs*.* 27. After the second injection, there was significant increase of the IgG responses against the *S. flexneri* serotypes only when rabbits were immunized 1 month after primary injection. No booster was observed for *S. sonnei*, either with 1 month or 3 months interval schedule. Comparing the responses elicited by GMMA post second dose, total IgG were higher with the 0–1 month schedule for all *Shigella* serotypes but *S. flexneri* 2a, for which IgG responses were similar (Figure 5A). SBA titers analysed in post-2 sera paralleled IgG responses, with exception of titers against *S. flexneri* 2a that were significantly higher at day 98 vs*.* day 42 (Figure 5B).

Similarly to what obtained with GMMA, pre-second vaccination similar anti-OAg specific IgG responses were elicited by glycococonjugates for all serotypes at day 27 (pre-second immunization at day 28) or 83 (pre-second vaccination at day 84), with exception of *S. sonnei* IgG that were higher at day 27. Second immunization with glycoconjugates always boosted the total IgG response irrespectively of the immunization scheme used, except for *S. flexneri* 2a with the 0–3 months protocol. Comparing the responses elicited by glycoconjugates post second dose, total IgG were similar with the 0-1 or 0–3 months schedule for all *Shigella* serotypes but *S. flexneri* 2a, for which IgG response was higher at day 42 (Figure 5C). However, antibody titers were not bactericidal against the *S. flexneri* serotypes when the second immunization was done 3 months after the first one (Figure 5D).

## 4 Discussions

*Shigella* is leading bacterial cause of diarrheal disease, more often associated with antimicrobial resistance (AMR) (Ranjbar and Farahani, 2019) and listed among those pathogens for which the development of new interventions is a global health priority by the WHO (World Health Organisation, 2022). To date, no vaccines are widely available against *Shigella*, but different OAg-based candidates are being evaluated in the clinic. The heterogeneous distribution of *Shigella* serotypes, across countries and overtime, determined by the OAg structural features, implies that multi-component vaccines are required to address the burden of shigellosis (Raso et al., 2023).

In this work we have compared GMMA and the more traditional glycoconjugation approach for a multi-component OAg-based vaccine against *Shigella*.

Sugar length and polysaccharide to protein ratio are two well known parameters that can affect the immunogenicity of glycoconjugates (Micoli et al., 2023). Both for *Shigella* glycoconjugates (Raso et al., 2020) and GMMA (Raso et al., 2020; Gasperini et al., 2021) we have previously verified no major role of OAg length on the ability to induce anti-OAg IgG antibodies with functional activity in mice. However, here, OAg populations used for conjugation to CRM_197_, a carrier protein commonly used for glycoconjugates (Micoli et al., 2018a), were quite similar in size to those displayed on GMMA. Major difference remained for *S. sonnei*, as GMMA also present a very long G4C polysaccharide that was not used for conjugation.

Moreover, *S. sonnei* GMMA and glycoconjugate were characterized by a similar OAg to total protein ratio, while the ratio was higher for *S. flexneri* GMMA than corresponding glycoconjugates. The impact of OAg density has been tested only with *S. sonnei* GMMA, finding that when compared at same OAg dose, GMMA with different number of sugar chains per total protein elicit a similar anti-OAg specific IgG bactericidal response (Rossi et al., 2023). However, the OAg to protein ratio could be critical for the immunogenicity of *Shigella* glycoconjugates (Micoli et al., 2023). Also to be considered that GMMA have a nanoparticle size and not only provide T-cell help to the OAg chains (Raso et al., 2020; Gasperini et al., 2021; Micoli et al., 2023), but also favour presentation of multiple OAg copies in their native bacterial environment. Furthermore, GMMA possess pathogen-associated molecular patterns, e.g. lipopolysaccharide and lipoproteins, that can provide self-adjuvanticity (Piccioli et al., 2022; Micoli et al., 2023). For this reason, GMMA and glycoconjugates were compared in this study with and without Alhydrogel.

Comparison between GMMA and glycoconjugates was based on anti-OAg specific IgG response and bactericidal titers. Many studies have demonstrated association between anti-OAg IgG titers and protection (Cohen et al., 2019) and more recently, by analysing serologic and vaccine efficacy data from two randomized vaccine-controlled trials of a *S. sonnei* conjugate vaccine conducted in young adults and children aged 1–4 years in Israel, a serum IgG anti-*S. sonnei* LPS threshold has been proposed as correlate of protection (Cohen et al., 2023). Also serum bactericidal activity has been proposed as important readout for *Shigella* vaccines (Ndungo and Pasetti, 2020).

In the absence of Alhydrogel, GMMA elicited significantly higher IgG antibodies than glycoconjugates, both after first and second vaccination in mice. Moreover, bactericidal activity of sera elicited by GMMA was higher in comparison to glycoconjugates. When adsorbed on Alhydrogel, serotype specific IgG responses became comparable but GMMA continued to elicit significantly higher SBA titers than glycoconjugates. Higher functionality could be linked to a broader IgG isotype switch observed with GMMA (Piccioli et al., 2023). These results are in agreement with those previously observed comparing *Salmonella* GMMA and glycoconjugates in mice (Micoli et al., 2018b).

In this study, we also compared for the first time the GMMA and glycoconjugate technologies in rabbits. Results obtained confirmed improved immune response elicited by *S. sonnei* GMMA with respect to glycoconjugate, despite presence or absence of Alhydrogel; while, differently from what observed in mice, *S. flexneri* GMMA and glycoconjugates elicited comparable immune responses.

To be noted that ELISA against *S. sonnei* was run by using LPS as coating antigen and we could not exclude quantification of higher levels of antibodies against core or lipid A from GMMA compared to glycoconjugate immunization. However, we had already verified by immunizing animals with OAg negative GMMA that these antibodies are not bactericidal (Mancini et al., 2021a).

Alhydrogel has been used in clinic as adsorbant to further reduce potential GMMA reactogenicity (Micoli et al., 2022b). Results from our studies suggest no need of Alhydrogel to increase GMMA immunogenicity, both in mice and rabbits. This could be expected due to the self-adjuvanting nature of GMMA, and actually an overstimulation by adding an adjuvant to already highly immunogenic GMMA might be detrimental for an optimal immune response. Viceversa, Alhydrogel works as adjuvant for the immunogenicity of glycoconjugates only in mice.

When *Shigella sonnei* and *S. flexneri* 2a glycoconjugates and *S. sonnei* GMMA have been tested as monovalent formulations in adults, no increase of the anti-LPS IgG response has been observed after a second injection with an interval of 4–6 weeks post first vaccination (Micoli et al., 2022b; Phalipon and Mulard, 2022). Here we compared in rabbits two different schedules, with second vaccination after one or 3 months after the first one, to understand if a longer interval could result in improved booster and higher response post-2. Both GMMA and glycoconjugates were able to elicit serotype specific antibodies that persist up to 3 months post vaccination. Glycoconjugates boosted the response both after one or 3 months for all serotypes, but antibodies were not functional when the second vaccination was given with a 0–3 months schedule. This could be related to a different quality and functionality of antibodies persisting at 3 months vs. 1 month post primary vaccination. Similarly to what seen in adults, no booster was observed for *S. sonnei* GMMA and this was independent from the immunization schedule used. An interval of 1 month resulted instead in increased response for *S. flexneri* GMMA after the second injection. Overall a longer interval of 3 months between vaccinations did not result in improved immunogenicity: all GMMA, but *S. flexneri* 2a, induced stronger functional IgG post second vaccination at 1 month vs*.* 3 months interval.

In conclusion, here we have compared two different approaches for the development of a multi-component OAg based vaccine against *Shigella*, both in mice and rabbits. Results obtained in the two animal models were different and it will be interesting to look at clinical data that will become available in the near future from different kind of OAg-based vaccines and in different age group populations to see if and how preclinical data can be predictive for humans.

## Data Availability

The original contributions presented in the study are included in the article/Supplementary Material, further inquiries can be directed to the corresponding author.
