# Supplementary material for: Comparison of Shigella GMMA and glycoconjugate four-component formulations in animals
Source: Front Mol Biosci. 2023 Nov 16;10:1284515. doi: 10.3389/fmolb.2023.1284515 (PMC10690372; doi:10.3389/fmolb.2023.1284515)
Supplement: Supplementary file 1 [file Table1.DOCX]

Supplementary Material

# Supplementary Tables

**Table S1.** Codes of secondary antibodies used in this study.

| **Secondary antibody** | **Code** |
| --- | --- |
| Rb anti-mouse IgG1 AP | Sigma SAB3701172 |
| Rb anti- mouse IgG2a AP | Sigma SAB3701179 |
| Rb anti- mouse IgG2b AP | Sigma SAB3701186 |
| Rb anti- mouse IgG3 AP | Sigma SAB3701193 |
| Gt anti- mouse IgG AP | Sigma A3438 |
| Rb anti- mouse IgM | Sigma A9688 |
| Anti-Rabbit IgG-AP | Sigma A3687 |
| Anti-Rabbit IgM-AP | Sigma AB97192 |

**Table S2.** Comparison of GMMA and glycoconjugates 4-component formulations in mice. CD1 mice were immunized i.p. at day 0 and 28 with 150 ng total OAg dose in absence or presence of 0.7 mg/mL of Alhydrogel (Al^3+^). Mann-Whitney two-tailed test was performed on IgG subclasses and IgM ELISA results at day 42: ns p > 0.05, * p < 0.05; ** p < 0.01; *** p < 0.001.

| **GMMA vs Conjugates  without Alhydrogel** | | **IgG1** | **IgG2a** | **IgG2b** | **IgG3** | **IgM** |
| --- | --- | --- | --- | --- | --- | --- |
| ***S. sonnei*** | Day 42 p value | ******* | ******* | ******* | ******* | ******* |
| ***S. flexneri* 1b** | Day 42 p value | ****** | ******* | ******* | ******* | ***** |
| ***S. flexneri* 2a** | Day 42 p value | ns | ******* | ******* | ******* | ***** |
| ***S. flexneri* 3a** | Day 42 p value | ***** | ******* | ******* | ******* | ns |
| **GMMA vs Conjugates  with Alhydrogel** | | **IgG1** | **IgG2a** | **IgG2b** | **IgG3** | **IgM** |
| ***S. sonnei*** | Day 42 p value | ***** | ns | ns | ******* | ns |
| ***S. flexneri* 1b** | Day 42 p value | ns | ns | ns | ns | ns |
| ***S. flexneri* 2a** | Day 42 p value | ns | ***** | ****** | ****** | ****** |
| ***S. flexneri* 3a** | Day 42 p value | ns | ns | ns | ***** | ns |

# Supplementary Figures


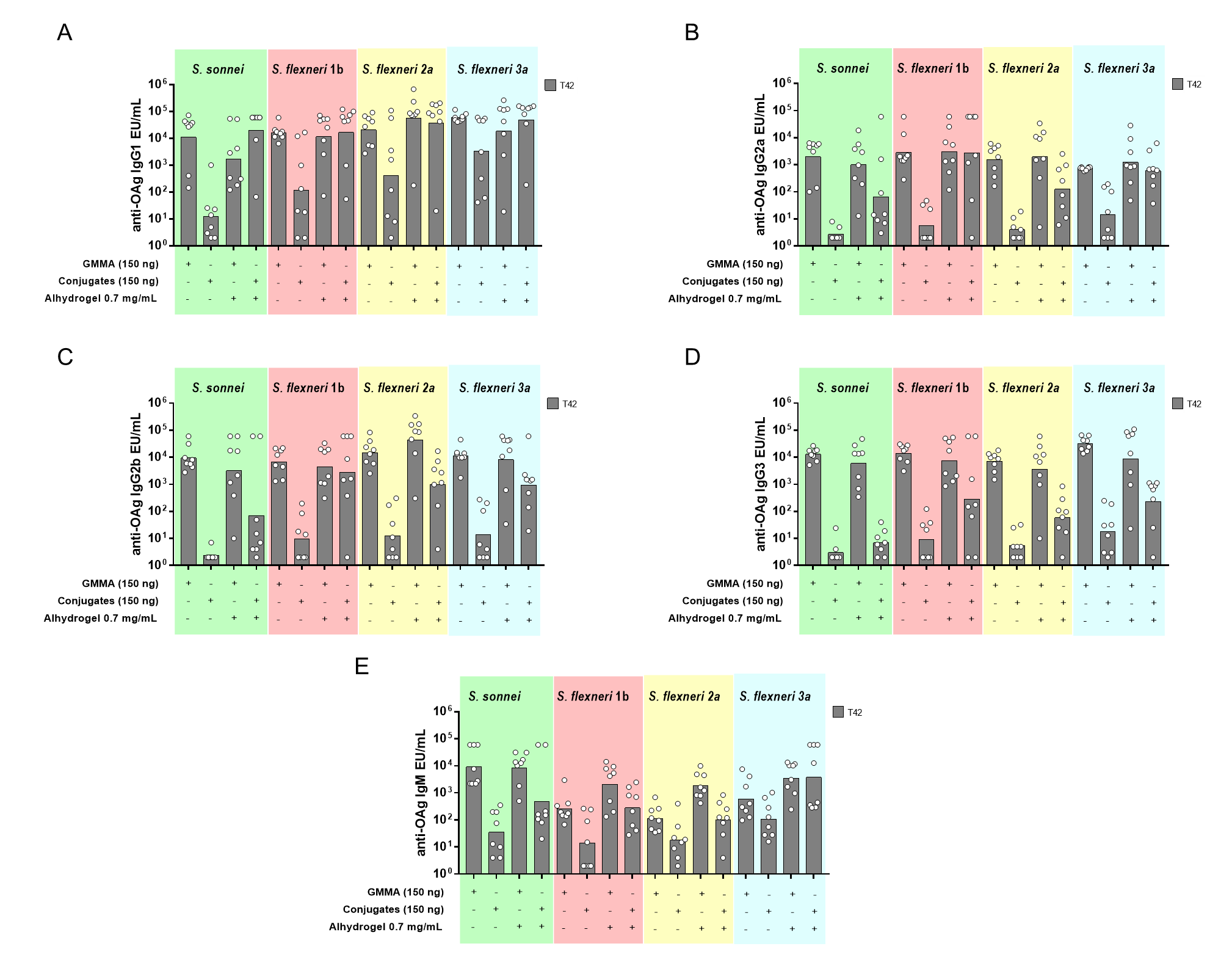


**Figure S1**. Characterization of the quality of the humoral response elicited by GMMA and glycoconjugates in mice. CD1 mice were immunized i.p. at day 0 and 28 with 150 ng total OAg per dose in absence or presence of Alhydrogel. **(A-D)** Anti-OAg-specific IgG subclasses and **(E)** IgM were evaluated at day 42. Summary graphs of geometric mean units (bars) and individual levels (dots) are reported.
